# AMPKα pathway involved in hepatic triglyceride metabolism disorder in diet-induced obesity mice following *Escherichia coli* Infection

**DOI:** 10.18632/aging.101623

**Published:** 2018-11-06

**Authors:** Jing Fang, Fengyuan Wang, Hetao Song, Zhengyi Wang, Zhicai Zuo, Hengmin Cui, Yiping Jia, Junliang Deng, Shumin Yu, Yanchun Hu, Liuhong Shen, Xiaoping Ma, Zhihua Ren, Liping Gou

**Affiliations:** 1 College of Veterinary Medicine, Sichuan Agricultural University, Chengdu, Sichuan 611130, PR China; 2 Sichuan Center for Animal Disease Control and Prevention, Chengdu, Sichuan 610041, PR China; 3 Heze Animal Husbandry and Veterinary Bureau, Heze, Shandong 274000, PR China; * Equal contribution

**Keywords:** Escherichia coli infection, hepatic triglyceride metabolism, AMPKα pathway, mouse

## Abstract

To investigate the different effects of acute pulmonary infection induced by *Escherichia coli* (*E. coli*) on lipid metabolism between diet-induced obesity (DIO, fed with high-fat diet) mice and lean mice. A total of 180 ICR mice were selected to be challenged intranasally with phosphate-buffered saline or 10^9^ CFUs/mL of *E. coli*, and the body character indexes, biochemical indexes and expressions of genes and proteins involved in lipid metabolism were examined pre- and post-infection. Results revealed that, before infection, DIO mice had significantly higher body weight, adipose and liver indexes, free fatty acid and triglyceride contents than lean mice. After infection, increased free fatty acid and triglyceride contents, increased expressions of resistin, SREBP-1c, ACC1, FAS and SCD-1, and declined PPARα, CPT-1α expressions and AMPKα phosphorylation were detected in the infected group, while the change rates were more serious in the lean mice than the DIO mice. The above-mentioned findings verified that, after being infected with *E. coli*, hepatic lipid metabolism disorder was aggravated by activating SREBP-1c related lipid synthesis pathway and inhibiting PPARα related fatty acid oxidation pathway. However, infection-induced lipid metabolic disorders was slighter in the DIO mice than the lean mice through AMPKα pathway.

## Introduction

With the improvement of living standards, nonalcoholic fatty liver disease (NAFLD) has become a global public health problem affecting approximately 15-20% of the general population in Asia and over 30% in West [1]. Obesity is an independent factor for NAFLD, which can increase the prevalence of NAFLD by 40%-50%, and almost 2/3 of obese patients are hepatic steatosis [2]. Excessive lipid accumulation, insulin resistance and low-grade inflammation in obesity were suggested to be involved with the development of NAFLD [3]. Additionally, infection and inflammation can cause lipid metabolism disorders. Patients with bacterial or viral infections and animals treated with lipopolysaccharide (LPS) or lipoteichoicacid (LTA) exhibited increased serum triglycerides levels and hepatic lipid accumulation [4]. Our preliminary studies found that obese mice exhibited improved host defenses against infection, and promoting recovery in *E. coli*-induced acute non-fatal bacterial pneumonia with enhanced immune response [5]. However, other studies showed that obesity was a risk factor for pneumonia [6], nosocomial infections [7], and surgical infections [8]. Therefore, the link between acute infection and obesity remains unknown, especially involving lipid metabolism.

Liver is the primary lipometabolic organ for fatty acid oxidation and *de novo* fatty acid synthesis, and the presence of NAFLD is associated with hepatic lipid metabolism disorders [9]. Hepatic lipid accumulation is developed as a result of abnormal fatty acid metabolism. Peroxisome proliferator-activated receptor α (PPARα) and sterol regulatory element binding proteins-1c (SREBP-1c) are two important factors known to regulate fatty acid oxidation and *de novo* fatty acid synthesis in liver by controlling the transcription of their downstream genes respectively. PPARα is a nuclear receptor that regulate lipid metabolism [10] and is also implicated in the development and intensity of inflammatory responses [11]. It modulates the gene transcription of CPT-1α, a rate-limiting enzyme of fatty acid oxidation [12]. SREBP-1c can partly regulate the expression of fatty acid synthase, lipoprotein lipase, and leptin genes [13]. To investigate the mechanism underlying *E.coli*-induced hepatic lipid metabolism disorder, the levels of PPARα and SREBP-1c and their downstream genes were measured. Furthermore, AMP-activated protein kinase (AMPK) is a serine/threonine kinase, functions as a critical energy sensor, integrates different signaling pathways to inhibit energy-consuming processes and to activate energy-producing processes. In the present study, basing on AMPKα- PPARα/SREBP-1c signal pathway, we compared hepatic lipid metabolism of lean and DIO mice with *E. coli* intranasal instillation, to determine whether acute infection could exacerbate lipid disturbance in obesity.

## RESULTS

### Lipid metabolic abnormalities in DIO mice

**Figure 1:**
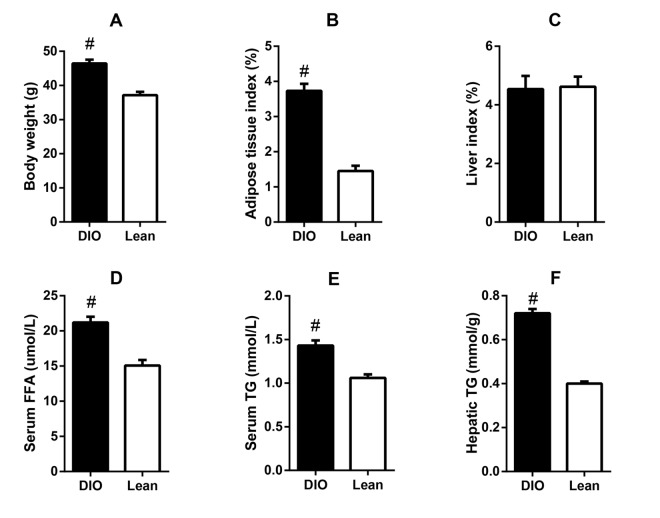
**Lipid metabolic abnormalities in the DIO mice.** (**A**) body weight, (**B**) adipose tissue index, (**C**) serum FFA level, (**D**) serum TG level, (**E**) liver index, and (**F**) liver TG content. Values are expressed as the mean ± SD (n=6); “#” indicates significant differences between the lean and DIO mice (p<0.05).

After feeding with high-fat diets for 8 weeks, the DIO mice exhibited significantly higher body weight and epididymal adipose tissue index than the lean mice, as well as higher serum concentrations of FFA and TG (p<0.05). Though the liver index showed no obvious changes, the liver TG content in the DIO mice was also markedly increased when compared with the lean mice (p<0.05) (Figure 1). Results suggested that the DIO mice displayed excessive accumulation of adipose tissue and lipid metabolic abnormalities.

### Differences in weight loss and adipose tissue lipolysis in mice following *E. coli* infection

**Figure 2:**
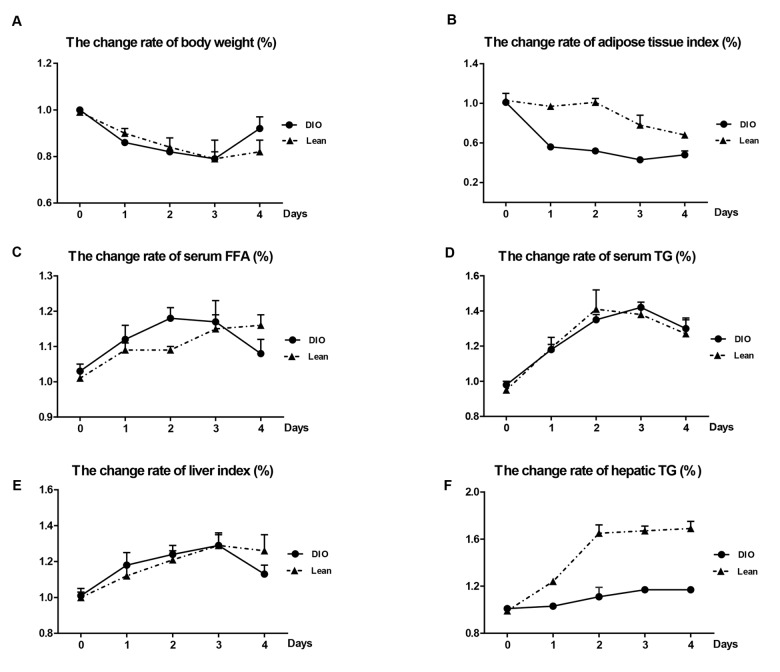
**Effects of *E. coli* infection on body weight and adipose tissue lipolysis in the lean and DIO mice.** (**A**) Body weight, (**B**) adipose tissue index, (**C**) serum FFA levels, (**D**) serum TG levels, (**E**) Liver index and (**F**) Hepatic TG contents. DIO indicates the ratio of DIO-infected/ DIO-uninfected, while Lean indicated the ratio of lean-infected/ lean-uninfected.

Following *E. coli* infection, compared with uninfected mice, the body weight in both the lean and DIO-infected mice were significantly decreased from 1 to 3 day post-infection (day post-infection = d.p.i), and increased at 4 d.p.i respectively (Table 1). Though the DIO mice had a higher total body weight than the lean mice during the infection, they exhibited greater weight loss from 1 to 3 d.p.i and recovered better at 4 d.p.i (Figure 2A).

**Table 1:** The body weight, organ indexes and free fatty acid and triglyceride contents.

Index	Groups	0d	1d	2d	3d	4d
Body weight (g)	DIO-infected	46.42±1.13^A^	40.77±0.56^A^	38.77±1.41^A^	37.95±2.04^A^	43.52±3.37^A^
	DIO-uninfected	46.49±0.54^A^	47.17±1.04^B^	47.53±1.45^B^	47.86±1.46^B^	47.27±1.43^B^
	Lean-infected	36.92±1.05^B^	33.52±1.41^C^	31.97±2.47^C^	30.05±3.9^C^	31.25±2.61^C^
	Lean-uninfected	37.15±0.96^B^	37.40±1.18^D^	38.00±1.27^A^	37.89±0.89^A^	38.04±0.76^D^
Adipose tissue index (%)	DIO-infected	3.73±0.2^A^	2.09±0.19^A^	1.93±0.16^A^	1.58±0.1^A^	1.8±0.26^A^
	DIO-uninfected	3.68±0.18^A^	3.72±0.16^B^	3.73±0.16^B^	3.66±0.14^B^	3.76±0.22^B^
	Lean-infected	1.49±0.25^B^	1.45±0.09^C^	1.52±0.12^C^	1.16±0.22^C^	1.04±0.08^C^
	Lean-uninfected	1.45±0.15^B^	1.5±0.09^C^	1.51±0.15^C^	1.48±0.09^A^	1.53±0.07^A^
Liver index (%)	DIO-infected	4.54±0.45^A^	5.33±0.55^A^	5.61±0.52^A^	5.83±0.77^A^	5.15±0.32^A^
	DIO-uninfected	4.50±0.47^A^	4.51±0.24^B^	4.54±0.57^B^	4.51±0.44^B^	4.63±0.71^B^
	Lean-infected	4.62±0.34^A^	5.28±0.42^A^	5.62±0.34^A^	6.08±1.00^A^	5.91±0.75^C^
	Lean-uninfected	4.64±0.44^A^	4.72±0.47^B^	4.68±0.44^B^	4.69±0.64^B^	4.67±0.38^B^
Serum FFA (umol/L)	DIO-infected	21.2±0.81^A^	23.64±0.95^A^	23.86±0.57^A^	22.43±0.49^A^	21.74±0.89^A^
	DIO-uninfected	20.58±0.99^A^	21.21±1.31^B^	20.19±0.98^B^	20.47±0.73^B^	20.88±1.19^A^
	Lean-infected	15.27±1.04^B^	17.05±0.8^C^	17.19±0.83^C^	17.86±0.48^C^	17.97±0.63^B^
	Lean-uninfected	15.07±0.79^B^	15.65±0.8^C^	15.76±0.83^D^	15.59±1.39^D^	15.53±0.77^C^
Serum TG (mmol/L)	DIO-infected	1.39±0.03^A^	1.62±0.11^A^	1.78±0.11^A^	1.85±0.18^A^	1.73±0.15^A^
	DIO-uninfected	1.43±0.06^A^	1.37±0.06^B^	1.33±0.09^B^	1.3±0.12^B^	1.33±0.05^B^
	Lean-infected	1.06±0.04^B^	1.21±0.08^C^	1.36±0.05^B^	1.45±0.1^B^	1.36±0.07^B^
	Lean-uninfected	1.12±0.08^B^	1.02±0.05^D^	0.97±0.1^C^	1.05±0.08^C^	1.08±0.12^C^
Hepatic TG (mmol/g)	DIO-infected	0.72±0.02^A^	0.74±0.02^A^	0.79±0.01^A^	0.85±0.07^A^	0.84±0.04^A^
	DIO-uninfected	0.72±0.03^A^	0.72±0.03^A^	0.71±0.06^B^	0.73±0.04^B^	0.72±0.02^B^
	Lean-infected	0.40±0.01^B^	0.50±0.04^B^	0.68±0.03^B^	0.71±0.05^B^	0.68±0.02^B^
	Lean-uninfected	0.40±0.01^B^	0.40±0.02^C^	0.41±0.03^C^	0.42±0.03^C^	0.40±0.03^C^

Notes: Values are displayed as mean ± SD (N=6). In the same column, values with different letter mean significant difference (P<0.05), while those with the same letter mean no significant difference (P>0.05).

In the DIO-infected mice, the epididymal adipose tissue indexes markedly declined during the whole experiment, and fell to the lowest point at 3 d.p.i, but only showed the decreased tendency in the lean-infected mice after *E.coli* infection (Table 1). The change rates of epididymal adipose tissue indexes of the DIO mice were more obvious than those of the lean mice at all-time points (Figure 2B).

The levels of FFA and TG in serum in the lean and DIO-infected mice were significantly increased during the infection compared with the lean and DIO-uninfected mice, respectively (Table 1). However, the change rates of serum FFA and TG in the DIO mice were more obvious compared with the lean mice before 3 d.p.i (Figure 2C) at 3 and 4 d.p.i (Figure 2D), respectively.

### Increased hepatic lipid accumulation in mice following *E. coli* infection

As shown in Table 1, the liver indexes and hepatic TG contents in both the lean and DIO-infected mice were significantly increased in response to *E.coli* administration, and reached peak at 3 d.p.i (p<0.05) and dropped at 4 d.p.i compared with each uninfected mice. The change rates of liver indexes following infection were increase in the DIO mice at 1 and 2 d.p.i, but obviously dropped at 4 d.p.i compared with the lean mice (Figure 2E). Compared with the lean mice at the same time point, the DIO mice exhibited higher hepatic TG contents, but the change rates of hepatic TG contents in the DIO mice were obviously lower than those of the lean mice after infection (p<0.05) (Figure 2F).

### Effects of *E.coli* infection on mRNA levels of hepatic lipid synthesis and oxidation related transcription factors in mice

**Figure 3:**
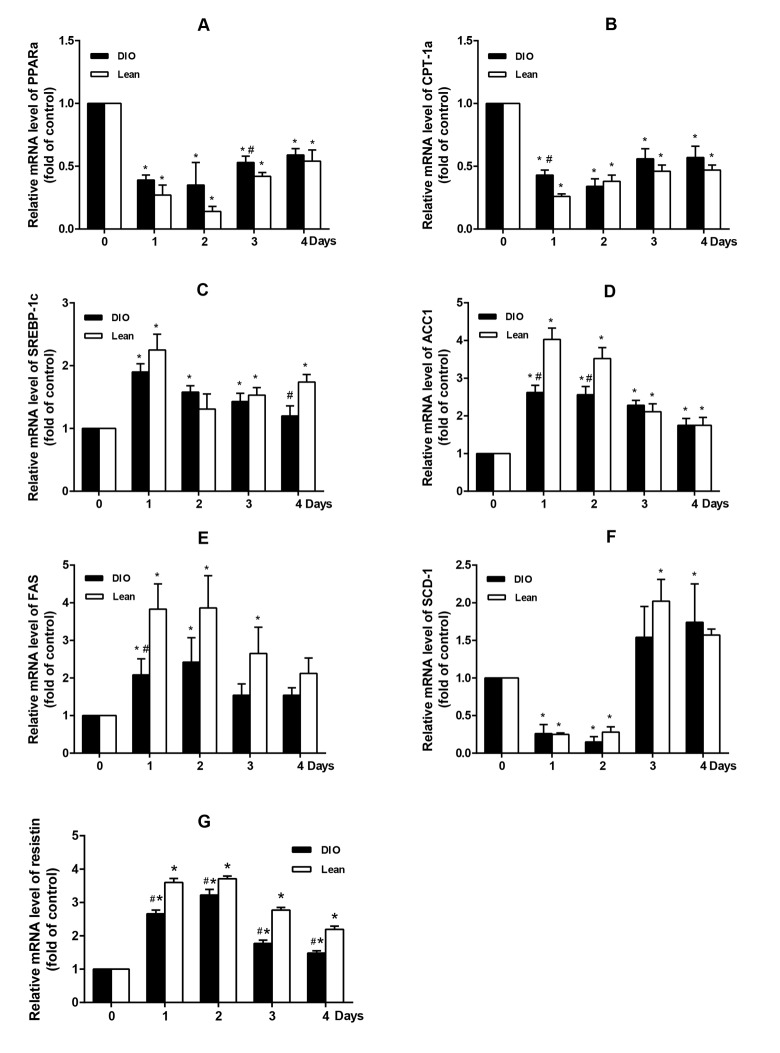
**Effect of *E. coli* infection on the mRNA expression of hepatic lipid metabolism related genes in the lean and DIO mice by qRT-PCR.** (**A**) PPARα, (**B**) CPT-1α, (**C**) SREBP-1c, (**D**) ACC1, (**E**) FAS, (**F**) SCD-1 and (**G**) Resisin. Values are displayed as mean ± SD (n=6). “*”p<0.05 compared with the control (0 d or pre-infection) within the time of infection; “#”p<0.05 between the lean and DIO mice at the same time point of infection.

From Figure 3, following *E.coli* infection, the mRNA levels of PPARα and its target gene CPT-1α in livers of both the lean and DIO-infected mice were significantly decreased from 1 to 4 d.p.i, and fell to the lowest points at 1 or 2 d.p.i compared with each uninfected mice (p<0.05). Moreover, the declined PPARα and CPT1α expressions in the DIO mice were slighter than those in the lean mice. (p<0.05).

The mRNA levels of SREBP-1c, ACC1 and FAS were significantly increased in both the lean and DIO-infected mice after infection and peaked at 1 or 2 d.p.i (p<0.05) when compared with the uninfected mice. However, the expression of SCD-1 markedly decreased from 1 to 2 d.p.i firstly, and then significantly increased from 3 to 4 d.p.i (p<0.05). Compared with the lean mice, the DIO mice mainly exhibited lower expressions of these genes. (p<0.05).

Compared with the uninfected mice, after being infected with *E.coli*, the mRNA level of resistin was significantly increased in both the lean and DIO-infected mice, and reached a peak at 2 d.p.i. Moreover, when compared with the lean mice, the expression of resistin in the DIO mice was relative low (p<0.05) (Figure 3G).

### Effects of *E.coli* infection on relative protein levels of hepatic lipid synthesis and oxidation related factors in mice

**Figure 4:**
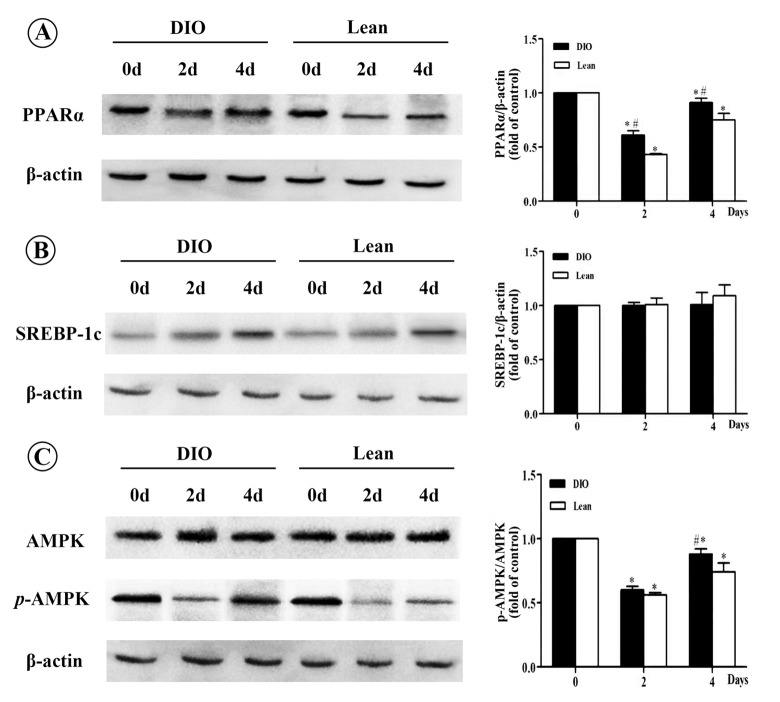
**Effect of *E. coli* infection on the protein levels of PPARα, SREBP-1c and *p*-AMPK/AMPK in liver of the lean and DIO mice by Western blotting.** (**A**) PPARα, (**B**) SREBP-1c, (**C**) *p*-AMPK/AMPK. Values are displayed as mean ± SD (n=6). “*”p<0.05 compared with the control (0 d or pre-infection) within the time of infection; “#”p<0.05 between the lean and DIO mice at the same time point of infection.

As shown in Figure 4, after *E. coli* infection, the relative protein levels of PPARα in both the lean and DIO-infected mice were significantly decreased at 2 and 4 d.p.i compared with each uninfected mice, and this decrease were significantly in the DIO mice lower than in the lean mice (p<0.05). However, the protein levels of SREBP-1c showed no obvious changes in response to *E.coli* infection (p>0.05). When compared with the uninfected mice, phosphorylation of AMPKα was notably decreased in both the lean and DIO-infected mice after being infected with *E.coli* (p<0.05), and this decrease was more obviously in the lean mice at 4 d.p.i than the DIO mice.

## DISCUSSION

Lipid metabolism disorder induced by inflammation or infection was characterized by increased plasma TG levels and accumulated hepatic lipid resulted from adipose tissue lipolysis, increased *de novo* hepatic fatty acid synthesis, and suppression of fatty acid oxidation [4]. As a risk factor of metabolic syndrome, obesity was also linked to the lipid metabolic disorder. The chronic low-grade inflammations were suggested to play an important role in the development of obesity-associated dyslipidemia [14]. However, there were few researches regarding the effect of acute infection on lipid metabolism in obesity. By the reports as to obese populations, obese patients had increased susceptibility to bacterial infection, especially bacterial lung injury [15]. Previous study also demonstrated that obese mice infected with *E. coli*-induced acute lung injury exhibited higher levels of TNF-α, IL-6 and IL-10 in serum [5]. In this study, the triglyceride metabolism of DIO mice following intranasal administration of 10^9^ CFUs/mL of *E. coli* was detected to determine the effect of gram-negative bacteria-induced acute lung injury on the lipid metabolic disorders in obesity.

After feeding with a high-fat diet for 8 weeks, the DIO mice had higher adipose tissue and liver index, serum FFA and TG contents, as well as hepatic TG contents, suggesting that the DIO mice exhibited excessive fat accumulation and dyslipidemia. After being infected with *E. coli*, the contents of serum FFA in both the lean and DIO-infected mice were significantly increased. Similarly, as reported previously, LPS-treated Zucker ^fat/fat^ mice had increased serum FFA levels for the increased lipolysis [16]. Moreover, circulating FFA could be taken up by tissues (muscle, heart, liver, etc.) for energy supplying, however, when the amount of FFA entering the liver was greater than its disposal, excess TG would be synthesized and secreted into blood or stored in liver, led to hypertriglyceridemia and hepatic steatosis [16]. Marangoni et al. reported that BALB/c mice displayed significantly increased serum TG contents in response to acute liver infection induced by intraperitoneal administration of Chlamydia pneumoniae [17]. Therefore, *E. coli*- induced acute pulmonary infection could aggravate the dyslipidemia in both the lean and DIO mice.

Adipose tissue is an important organ of lipid deposition which can be recruited in times of need to provide fuel for other organs [18]. Following *E. coli* infection in this study, the DIO-infected mice experienced greater loss of adipose tissue index from 1 to 4 d.p.i, indicating that stronger fat mobilization was taken place in the DIO mice than the lean mice. Liver is the central organ of lipid metabolism that maintains balance between lipid availability and lipid disposal [19]. Long-term ethanol consumption, infection or other specific etiologies might induce hepatic lipid accumulation [20-22]. Chen et al. reported that intraperitoneal injections of LPS for 24h was able to increase the liver weight and hepatic TG contents in CD-1 mice [23]. Moreover, chronic infection caused by continuous injection of LPS for 12 weeks had also determined to accelerate hepatic steatosis in high-disaccharide fed Zucker ^fat/fat^ mice [24]. In the present study, following *E.coli* infection, although the actual levels of hepatic TG in the DIO mice were higher than those in the lean mice, the change rates of hepatic TG contents were less in the DIO mice than the lean mice. As is known, triglycerides are the main constituents of body fat in humans and other animals, and liver cells is a place to synthesize and store triglycerides [25]. As a composition of adipose tissue, following the decrease of the adipose tissue index, the amount of hepatic TG might be declined, however, as infection induced hepatic lipid accumulation, the hepatic TG would increase. In our results, infection induced hepatic TG increasing was greater than fat loss induced hepatic TG decreasing, while it still play a role. Thus, the increasing rate of hepatic TG contents was slighter in the DIO mice than the Lean mice. These findings were similar to Zhang’s research [26], which indicated that high-fat diet-induced obesity might have increased tolerance to acute inflammation. Nevertheless, the mechanisms remain unclear.

Infection and inflammation induced fatty acids oxidation decreasing and lipid synthesis enhancing are regulated by several transcriptional factors and enzymes, like PPARα and SREBP-1c [27]. To clarify the underlying mechanism, these transcriptional factors and enzymes were measured. Previous studies reported that infection and inflammation increased the expression of SREBP-1c and its target genes, like FAS and ACC, while inhibited the expression of PPARα *in vivo* and *in vitro* [17,28-30]. Also, high-fat diet-induced hepatic steatosis in obese mice were associated with inhibited PPARα activity and increased expressions of SREBP-1c, as well as its target genes, FAS and SCD-1 [31,32]. In addition, infection and inflammation could also modulate hepatic FFA oxidation and synthase. In the present study, 10^9^ CFUs/mL *E. coli* could up-regulate the expression of hepatic SREBP-1c, ACC1 (one subset of ACC) and FAS, and down-regulate the expression of PPARα and CPT-1α, which was accordance with LPS-induced infection in CD-1 mice [23]. These results suggested that *E.coli* infection aggravated hepatic triglyceride accumulation in the lean and DIO-infected mice, which at least partially attributing to the inhibition of hepatic FFA oxidation and activation of *de novo* fatty acid syntheses. However, it should be highlighted that we expected classical increase of SCD-1 mRNA level as SREBP-1c increased, however, SCD-1 mRNA level declined markedly at 1 and 2 d.p.i, and then increased significantly at 3 and 4 d.p.i. SCD-1 is a kind of endoplasmic reticulum enzyme that catalyzes the saturated fatty acid to form a single unsaturated fatty acid, providing substrates for the synthesis of TG [33]. The inconsistency between SREBP-1c and SCD-1 mRNA level may partly attribute to the other regulators, such as leptin, which can directly regulate the expression of SCD-1 by mechanisms independent of SREBP-1c [34], or monounsaturated fatty acid (MUFA) derived from extrahepatic tissues, which can increase the hepatic lipid accumulation without the effect of SCD-1 [35]. Moreover, in the present study, the expression of resistin showed a more tremendous increase in the lean mice than the DIO mice after infection. Resistin is an adipose-derived hormone, which was first identified in mouse adipose tissue as a negatively regulated PPARs responsive gene [36,37]. In the setting of obesity, resistin acted as adipocytokine and possessed proinflammatory property [38]. Above all, resistin played an important role in the process of lipid metabolism and inflammation in obesity. Moreover, Seo et al. reported that ADD/SREBP1c control resistin gene expression upon nutrition regulation and adipogenesis [37]. Thus, the lean mice exhibited greater inhibition effect of the PPARα expression than that of the DIO mice, and showed sharper increase of SREBP-1c expression under the control of resistin.

Activated AMPKα could inhibit the expression of SREBP-1c [39] and up-regulate the transcription levels of PPARα in liver [40]. High-fat diet-induced hepatic steatosis in obesity was associated with decreased AMPKα activity and disturbed PPARα and SREBP-1c related lipid metabolism signaling pathway [39,41]. In the present study, the ratio of p-AMPKα/AMPKα was decreased more slightly in response to *E. coli* infection in the DIO mice than in the lean mice, which was consistent with the changes of SREBP-1c and PPARα. Similarly, Andreasen et al. also reported that intravenous bolus of LPS had no significant influence on AMPKα phosphorylation in skeletal muscle between type II diabetes patients and normal ones [42].

**Figure 5:**
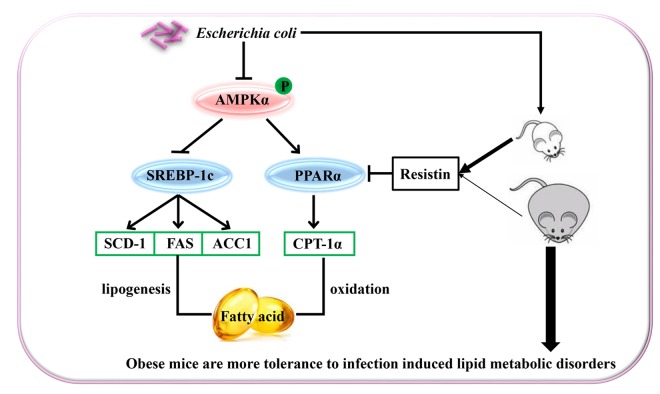
Schematic representation of *Escherichia Coli* induced hepatic triglyceride metabolism disorder in the lean and diet-induced obese mice through AMPKα pathway.

In summary, *E. coli*-induced acute lung infection aggravated dyslipidemia in mice by inhibiting PPARα related fatty acid oxidation pathway and activating SREBP-1c related lipid synthesis pathway, which resulted from the decreased phosphorylation of AMPKα. *E. coli*-induced change rate of lipid metabolism-associated parameters was slighter in the DIO mice than the lean mice, which may be partially related to the effect of resistin. These suggested that obesity could improve host tolerance to acute infection-induced lipid metabolic disorders (the proposed mechanisms showing in the Figure 5).

## MATERIALS AND METHODS

### Animals

21 to 28-day-old male ICR mice were purchased from Dashuo Biological Technology Company (Chengdu, China). The mice were kept in specific free-pathogen conditions and received either a normal or high-fat diet for 8 weeks, by which Lean and DIO mice could be obtained [43]. Both of normal and high-fat diets were purchased from Dashuo (Chengdu, China). According to the standard of human obesity, DIO mice were considered when the obese index was over 20% by the following formula [44].

$$Obese\;index=\frac{Individual\;weight\;of\;DIO-Average\;weight\;of\;Lean}{Average\;weight\;of\;Lean}\times 100\%$$

Then, the DIO and Lean mice were subdivided into 4 groups, namely DIO-infected, DIO-uninfected, lean-infected and lean-uninfected, respectively. The infected mice were anesthetized with ether and challenged intranasally with 40μL of bacterial suspension containing approximately 10^9^ CFUs/mL of *E. coli* diluted in PBS, and the uninfected mice were given same amount of PBS by the same way. *E. coli* was supplied by the Sichuan Agricultural University Veterinary Medical Laboratory. Preliminary study determined that 10^9^ CFUs/mL *E. coli* was able to elicit acute lung infection without any mortality in either DIO or Lean mice [5]. Food and water for mice were supplied *ad libitum*. The animals were used under protocols approved by the Guidelines for the Care and Use of Laboratory Animals and the Ethics Committee of Sichuan Agricultural University (Approval No: 2012-024).

### Body weight and organs index

At indicated time points, 0 d (pre-infection) and 1 d, 2 d, 3 d, 4 d (post-infection), body weight of mice were measured. Then mice were anesthetized with ether and euthanized, epididymal adipose tissue and liver were collected and weighed. Organ index was calculated by the following formula:

$$Organ\;index=\frac{Organ\;weight\;(g)}{Body\;weight\;(g)}\times 100\%$$

### Serum triglycerides and free fatty acid analyses

Blood samples were obtained retro-orbitally and individual sera were separated via centrifugation and stored at -20°C. Triglycerides and free fatty acid concentrations in the serum were measured with a commercially available kits (Nanjing Jiancheng Bioengineering Institute, China), according to the manufacturer’s instructions.

### Hepatic triglycerides analyses

Liver samples were homogenized in ice-cold phosphatic buffer solution according to the ratio of weight (g): Volume (ml) = 1:9, 2500r/min, centrifuge for 10 minutes to obtain the supernatant solution. Hepatic triglycerides content was determined using a commercially available kit (Nanjing Jiancheng Bioengineering Institute, China).

### RNA isolation, reverse transcription and quantitative real-time PCR

Total RNA was extracted from the liver by TRIzol reagent (Invitrogen) according to the manufacturer’s recommendations. The RNA concentration was determined spectrophotometrically on a Nano Drop 1000 spectrophotometer (Thermo Fisher Scientific, Waltham, MA, US). One microgram of total RNA was reverse transcribed into cDNA using standard reagents (Invitrogen). Quantitative real-time PCR was performed with SYBR Premix Ex TaqTM II kit (TAKARA BIO Inc) using the following primer (Table 2). The amplification reactions were carried out with an initial step (95°C for 30 s, 1 cycle) and the second step (95°C for 5 s and 60°C for 30 s, 39 cycles), finally, specific transcripts were confirmed by melting-curve profiles (cooling the sample to 65°C and heating slowly to 95°C with measurement of fluorescence). Relative gene expression was defined as a ratio of target gene expression versus β-actin gene expression. The 2^-ΔΔCt^ method was used to calculate the relative expression levels in mRNA abundance.

**Table 2:** Primers used for RT-PCR.

Items	Sense (5’-to- 3’)	Antisense (5’-to- 3’)
RETN	CTTCCTTGTCCCTGAACTGC	ACGAATGTCCCACGAGCC
PPARα	GAAGCTTTGGTTTTGCAGACT	GTCCCCACATATTCGACACTC
CPT-1α	CGAAGAACATCGTGAGTGG	GACGTCTGGAAGCTGTACAAC
SREBP-1c	CTTCTGGAGACATCGCAAAC	GGTAGACAACAGCCGCATC
ACC1	GGCAGCAGTTACACCACATAC	TCATTACCTCAATCTCAGCATAGC
FAS	GCACAGAAGGGAAGGAGT	CCAGGAGAATCGCAGTAG
SCD-1	CTGACCTGAAAGCCGAGAA	GTGAGACCAGTTGCG

### Western blotting

Hepatic total lysates were separated by SDS-PAGE and transferred on to a nitrocellulose membrane in an electrophoretic transfer cell (Bio-Rad, USA). At room temperature, the membranes were washed by TBST and blocked with 5% skimmed milk, then incubated with the following antibodies: anti-*p*-AMPKα-Thr172, anti-AMPKα, anti-SREBP-1c, anti-PPARα, anti-β-actin (Cell Signaling, Danvers, MA, USA) overnight at 4°C. After incubated with horseradish peroxidase-conjugated secondary antibody (Boster Bio-Engineering Co., Ltd), the membranes were washed and detected using diaminobenzidine (DAB) reagent (Tiangen, China).

### Statistical analysis

The significance of difference was analyzed by variance analysis, and results were presented as mean ± standard deviation (M ± SD). And Two-tailed, unpaired Student's t test was used to assess significance, with p<0.05 considered statistically significant. The change rate was calculated by the following formula, and DIO and Lean in the figure 2 indicated the change rate of the lean and DIO mice, respectively.

Change rate (%) = value of infected mice/value of uninfected mice ×100%
